# Shifu‐Inspired Fungal Paper Yarns

**DOI:** 10.1002/advs.202511975

**Published:** 2025-08-04

**Authors:** Anne Zhao, Mitchell P. Jones, Kathrin Weiland, Alexander Bismarck

**Affiliations:** ^1^ Polymer and Composite Engineering (PaCE) Group Institute of Material Chemistry and Research Faculty of Chemistry University of Vienna Währinger Straße 42 Vienna 1090 Austria; ^2^ Shaping Matter Lab Faculty of Aerospace Engineering Delft University of Technology Delft 2629 HS Netherlands; ^3^ Department of Chemical Engineering and Ecotoxicology University of Applied Sciences Technikum Wien Vienna 1090 Austria; ^4^ Division of Materials Science Department of Engineering Sciences and Mathematics Luleå University of Technology Luleå SE‐97187 Sweden

**Keywords:** biorefinery, fungal chitin, paper yarn, shifu, textiles

## Abstract

Fungal biorefinery is a popular method for producing advanced fabrics but is currently limited to leather alternatives and similar by the sheet‐based nature of most fungal materials. Biopolymers in the fungal cell wall, such as chitin and chitosan, are only soluble in harsh chemicals, making established extrusion‐based yarn production systems expensive and hazardous. The Japanese art of Shifu is used to produce fungal chitin‐β‐glucan yarns of varying linear density from engineered fungal sheets, enabling the production of yarns. Yarn mechanical strength is influenced by sheet precursor grammage and can be tuned using various chemical modifications such as glycerol‐based plasticization. Yarns hybridized with nanocellulose exhibited low strength, stiffness, and ductility, due to weak interfacing with fungal sheets. With mechanical properties outperforming commercial cellulose paper yarns and on par with cotton and viscose yarns, fungal yarns produced from engineered sheets of fungal biomass using Shifu techniques represent a viable yarn candidate for a broad range of applications, yet unachieved using fungi, such as textiles, upholstery, and carpets for the fashion and décor sectors.

## Graphical abstract

Fungal chitin‐β‐glucan sheets, processed using Shifu techniques, yield yarns with tunable strength and mechanical properties matching or surpassing cotton and viscose. Chemical modification and hybridization routes enable further property control. This approach positions engineered fungal biomass as a scalable, sustainable feedstock for advanced textile, upholstery, and decor applications.

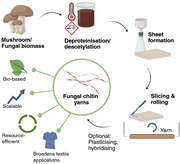

## 1 Introduction

Fungal biorefinery for the manufacture of sustainable materials with unique and useful properties has captured the imagination of academics, industry, and the public alike.^[^ ^1^ ^]^ Fungi derive their value as materials from the structural polymers that they contain, such as chitin. Chitin is a strong but brittle polymer prominently known as the primary constituent of crustacean exoskeletons and insect cuticles. It also exists in fungal cell walls, but unlike in crustaceans, here it is associated with β‐glucan, which renders the material more elastomeric. Fungal cell walls also contain chitosan, a partially deacetylated derivative of chitin, which reinforces the cell wall.^[^ ^2^ ^]^ This combination of polymers endows fungal biomass with unique and tunable mechanical properties, making them useful for materials.^[^ ^3^ ^]^

Fungi‐derived leather alternatives represent one of the more recent fungal materials. These materials rapidly gained attention and are now touted as popular candidates for advanced new fabrics.^[^ ^4^ ^]^ Various fermentation and sheet processing options exist to produce fungi‐derived leather alternatives, each providing variations in manufacturing parameters, mechanical, aesthetic, and haptic properties.^[^ ^5^ ^]^ However, the use of as‐grown fungal mycelium to create clothing and apparel is, to date, limited by the fact that the product is a sheet with size constraints and poor mechanical properties attributable to the heterogeneous nature of mycelium mats.^[^ ^5^ ^]^ This greatly restricts the products that can be produced from fungi in the fashion sector, as most garments are spun, woven, knitted or similar made from yarns.^[^ ^6^ ^]^ If fungal biopolymers could be shaped into yarns rather than sheets, fungal biomass may represent an attractive and sustainable source of fibers for the production of clothing.^[^ ^2a^ ^]^ Fungal yarns could provide an alternative to fossil‐derived and non‐biodegradable synthetic polymer fibers, such as polyesters.^[^ ^7^ ^]^ This alternative would only supplement rather than replace synthetic yarns. Polyester, the most common synthetic fiber, had an annual production volume of 63 Mt in 2022,^[^ ^8^ ^]^ which would be unattainable using fungi.

Some progress has been made toward producing chitinous monofilaments through wet spinning of fungal microfibers achieving tensile strength upto 69.5 MPa and Young's Moduli of 4.97 GPa.^[^ ^9^ ^]^ Multifilament fibers can also be wet spun from chitin and chitosan extracted from shellfish^[^ ^10^ ^]^ exhibiting tenacities of 6.1–45 cN/tex. A potential simpler solution for fungal yarns exists in the ancient Japanese art of Shifu, which experienced a resurgence in popularity for clothing production in the 20th century.^[^ ^11^ ^]^ Shifu describes the process of slicing paper into long strips, moistening and rolling it, forming a continuous thread, which is spun and eventually twisted in the moist state.^[^ ^12^ ^]^ Traditionally, “washi” paper comprising long, strong fibers derived from plant biomass, such as kōzo (*Broussonetia papyrifera*), mitsumata (*Edgeworthia chrysantha*), or gampi (*Wikstroemia trichotoma*), is cut into strips with a width of 1–3 mm using a sharp knife or blade.^[^ ^13^ ^]^ The strips are then crumpled and slightly moistened to reduce the paper stiffness, making it more pliable and easier to spin. Softened paper strips can be twisted between the fingers or rolled over a rough surface, such as a concrete block, with additional strips added over the course of this process by overlapping the ends of the paper strips and twisting them together to produce a continuous yarn.^[^ ^14^ ^]^

Paper yarns exhibit similar tenacity and strain‐to‐failure ε_*f*_ to common natural clothing yarns, such as cotton, similar tenacity but lower ε_*f*_ than viscose and lower tenacity and ε_*f*_ than polyester.^[^ ^15^ ^]^ Cellulose paper yarns also exhibit low shrinkage after washing (≤5%) compared to viscose (19–26%) and cotton (12–15%) yarns.^[^ ^15^ ^]^ The appearance and strength of paper yarns vary based on the quality of paper from which they are produced, however, they have been described as superior to hemp and jute.^[^ ^11a^ ^]^ Notably, since paper yarns are produced from (homogeneous) engineered paper, they do not exhibit biological variations common in other naturally formed fibers.^[^ ^11b^ ^]^

**Figure 1:**
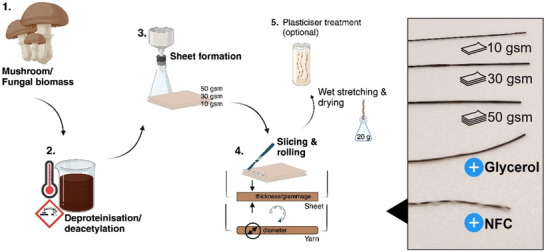
*Agaricus bisporus* mushrooms were macerated, deproteinized using 1 m NaOH and optionally deacetylated using 12 m (40%) NaOH. Sheets with varying grammage Γ (g m^−2^) were produced, sliced and rolled using the Shifu technique. Modified mechanical properties were achieved through plasticization using glycerol or hybridization with nanocellulose. Created in BioRender. Bismarck, A. (2025) https://BioRender.com/dpty2p7.

Despite rapidly increasing interest in fungal chitin,^[^ ^16^ ^]^ especially for textiles,^[^ ^9^ ^]^ a satisfactory yarn candidate derived from fungal biomass has yet to be achieved. Herein, we report the development and realization of fungal yarns of varying linear densities that could expand the applications of fungal biopolymers in the fashion sector beyond the current trend of leather alternatives. Fungal sheets of varying grammage were produced using standard papermaking procedures and sliced in the wet state (**Figure** **1**). Sliced strips were then processed into yarns using the Shifu technique, and their chemical, physical and mechanical properties characterized. We adapted paper yarn spinning^[^ ^11b^ ^]^ to conceptualize a manufacturing sequence for fungal materials and produce a new product, for which no other fungi‐derived candidate materials with competitive mechanical properties exist.

## 2 Results and Discussion

*A. bisporus* (white button) mushrooms were macerated, deproteinized and then optionally deacetylated prior to elemental and carbohydrate analysis and calculation of the chitin and chitosan content. Deproteinization of fungal fruiting bodies and subsequent deacetylation of deproteinized extracts were associated with dry yields of 13% and 3%, respectively, as compared to a dry weight of ≈14% for raw *A. bisporus* mushrooms.

**Figure 2:**
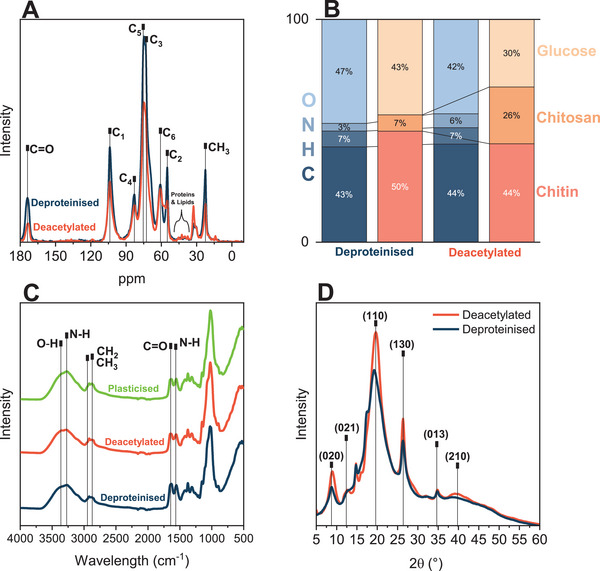
Chemical composition of macerated fungal biomass deproteinized using 1 m NaOH and optionally deacetylated using 12 mol L^−1^ (40 wt.%) NaOH. A) ssNMR spectra of deproteinized (blue) and deacetylated (red) extracts exhibit all significant C1–6 signals associated with the polysaccharide backbone. B) Elemental composition of deproteinized (blue) and deacylated (red) extracts. Glucosamine content represents the sum of the chitin and chitosan content, calculated based on the degree of acetylation (ssNMR: C═O and CH_3_ peak integrals). C) ATR‐FTIR spectra of deproteinized (blue), deacylated (red), and plasticized (green) yarns exhibiting all peaks associated with amine I and II bands, in addition to the functional groups (─OH, ─CH_2_ and ─C═O) contained in the structural biopolymers. D) X‐ray (WAXD) diffractogram of deproteinized (blue) and deacylated (red) extract exhibiting an amorphous halo likely from β‐glucan and significant peaks associated with α‐chitin or chitin‐β‐glucan complex (unassigned).

Alkaline treatments not only deacetylated chitin in the extract but also partially hydrolysed the carbohydrate backbone and cleaved glucan. ssNMR spectra of both deproteinized and deacetylated extracts (**Figure** **2**A) exhibited C1–6 signals between 55 and 104 ppm associated with the polysaccharide backbone found in chitin, chitosan, and glucan. Deacetylated extracts produced by boiling the macerated deproteinized fungal biomass in 12 m NaOH had considerably lower ─CH_3_ and C═O signals at 22.5 and 173.5 ppm, respectively than deproteinized extracts. The acetyl groups were removed by base‐catalyzed hydrolysis. The elemental analysis of this deacetylated fungal biomass extract showed considerably higher N content (6 wt.%) than that of the deproteinized extract (Figure 2B). Despite the mild nature of the alkaline treatment utilized for deproteinization, both fungal biomass extracts were partially deacetylated; deproteinized extracts exhibited a degree of acetylation DA = 87% and deacetylated extracts a DA = 63%. DA coupled with chitin/chitosan‐associated glucosamine and chitin‐linked‐glucan‐associated glucose carbohydrate content indicated a composition of 50% chitin, 7% chitosan and 43% β‐glucan in deproteinized extracts and 44% chitin, 26% chitosan and 30% β‐glucan in deacetylated extracts. These compositions resembled literature values utilizing similar alkaline treatments.^[^ ^17^ ^]^

ATR‐FTIR spectra indicated amide I and II bands associated with C═O stretching at 1663 cm^−1^ and N─H deformation at 1557 cm^−1^, respectively (Figure 2C). Both vibrations were more pronounced in deproteinized than deacetylated fungal yarns due to their higher degree of acetylation. Bands corresponding to N─H and O─H stretching were visible at 3283 and 3356 cm^−1^, respectively, for both yarns. ─CH_2_ and ─CH_3_ bands at 2920 and 2885 cm^−1^, C─OH stretching bands at 1153 cm^−1^, C─OH stretching bands at 1026 cm^−1^ and C─O─C bands at 1153 cm^−1^ were also present in both fungal yarns and are attributable to the backbone of the structural biopolymers. No significant change in H‐bonding could be observed between deproteinized, plasticized and deacetylated yarns when analyzing C─OH bands at 3356, 1153, and 1026 cm^−1^ (Figure S2, Supporting Information). The presence of water could affect the band associated with N─H deformation at 1557 cm^−1^. Figure S2b (Supporting Information) indicates a minimal broadening of the bands at 1663 and 1557 cm^−1^, with incorporation of glycerol, which could be due to increased hydrogen bonding to water as well as to glycerol.^[^ ^18^ ^]^

Despite a clear crystalline component in the deproteinized and deacetylated fungal extracts, the presence of a halo in both XRD diffractograms indicated the presence of amorphous components (Figure 2D). Transmission diffractograms exhibited diffraction peaks associated with α‐chitin: 020 (2θ = 9°), 021 (12°), 110 (19°), 130 (26°), 013 (32°) and 210 (39°). Since the chemical extract contains β‐glucan, it can be assumed that the unassigned peaks at 14.8°, 16°and 17.1° could be part of the β‐glucan diffractogram.^[^ ^19^ ^]^ The peak shape of the unassigned peaks also appears sharper with a smaller full width at half maximum (FWHM) than peaks associated with α‐chitin. The 2θ peak at 26.3° is assigned to the (130) of α‐chitin. However, the peak's small FWHM suggests that other components contribute to its position.^[^ ^20^ ^]^

In sheets produced from deproteinized chitin‐β‐glucan extracted from fungal biomass we observed microstructure features, which comprised assembled nanostructures. The microstructures consisted of collapsed fungal cell walls (hyphae) acting as carriers for chitin nanofibrils, as shown in the SEM micrographs (Figure S1, Supporting Information). Fungal sheets comprising deproteinized chitin‐β‐glucan were ≈33% stiffer and ≈62% stronger than those produced from deacetylated extracts (**Table** **1**). Chitin's acetyl amine group, which is comparatively “bulkier” than its deacetylated counterpart, may hinder the formation of favorable inter‐fiber hydrogen bonding; however, the branched glucan appears to compensate for this shielding effect, acting as an adhesive and enhancing structural toughness.^[^ ^21^ ^]^ The poorer mechanical properties associated with deacetylated fungal sheets may be attributable to the extract's lower glucan content, which results in reduced site availability for hydrogen bonding between fibers. Deacetylated sheets also exhibited higher porosity than deproteinized chitin‐β‐glucan sheets, which considerably influenced mechanical properties.

**Table 1:** Thickness *t*, envelope ρ_*e*_ and skeletal ρ_*s*_ density, porosity Φ, elastic modulus *E*, tensile strength σ, and strain‐to‐failure ε_*f*_ of 30 g m^−2^ (grams per square meter, gsm) sheets produced from deproteinized and deacetylated fungal biomass extracts and NFC sheets of the same grammage.

Sheets	*t* [µm]	ρ_*e*_ [g cm−^3^]	ρ_*s*_ [g cm^−3^]	Φ [%]	*E* [GPa]	σ [MPa]	ε_*f*_ [%]
Deproteinized chitin‐β‐glucan	40 ± 1	0.94 ± 0.07	1.50 ± 0.07	37.3 ± 5.5	5.5 ± 0.6	49.8 ± 4.2	1.3 ± 0.2
Deacetylated (chitosan)	30 ± 2	0.82 ± 0.05	1.48 ± 0.07	44.5 ± 2.6	4.1 ± 0.3	30.7 ± 1.4	1.2 ± 0.1
Nanofibrillated cellulose	40 ± 2	0.70 ± 0.02	1.60 ± 0.04	56.4 ± 1.7	3.1 ± 0.5	22.3 ± 3.0	1.2 ± 0.3

Elastic modulus *E* and tensile strength σ for NFC sheets produced from dried filter cake were less than half of those associated with their deproteinized chitin‐β‐glucan sheet counterparts when loaded in tension. The poor mechanical properties of nonactivated NFC sheets may be attributed to their high porosity due to poor interfibrillar bonding, which is a result of poor consolidation during air drying under 5 kg‐force. Attempts using hot pressed and densified NFC sheets resulted in sheets that proved to be too stiff and brittle to allow further processing and in lack of adhesion between NFC and chitin‐β‐glucan sheets, making hybridization impossible. Thus, we intentionally used the “as‐filtered” wet NFC filter cakes to produce Shifu‐like yarns. NFC sheet porosity (56%) resembled literature values for 50:50 steam exploded bamboo microfiber‐NFC papers (57%)^[^ ^22^ ^]^ and nanocellulose created from disintegrated recovered paper with high (60 min) pulping times (56%)^[^ ^23^ ^]^ and was lower than values for short (10 min) pulping times (79%)^[^ ^23^ ^]^ and bacterial cellulose nanopapers (67%).^[^ ^24^ ^]^ All sheets exhibited similar ε_*f*_ of 1.2 to 1.3%.

**Figure 3:**
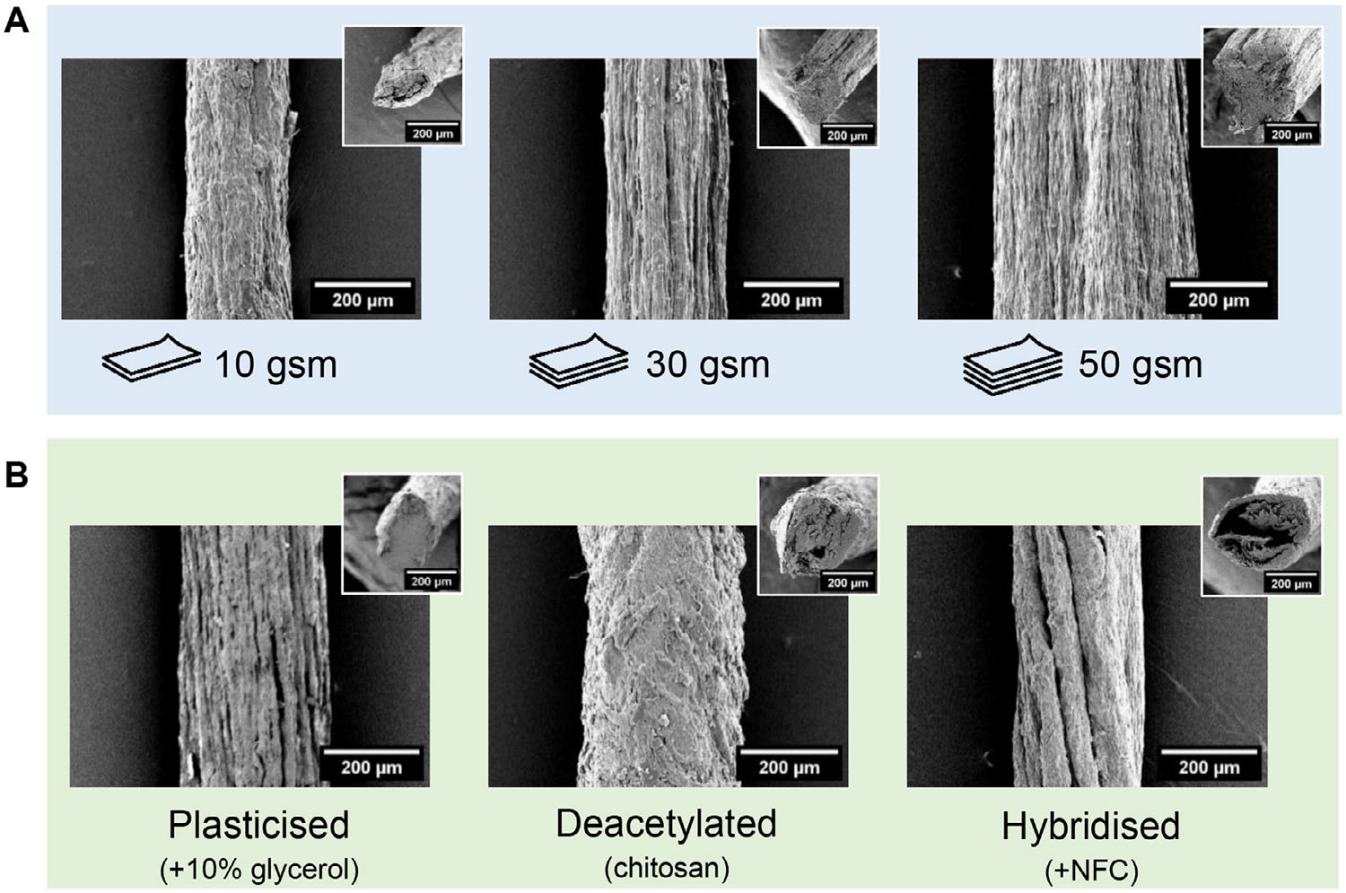
A) Characteristic SEM images (side view and cross‐sectional (inset) morphology) of yarns with varying linear density produced by slicing and rolling of deproteinized chitin‐β‐glucan sheets of different grammages Γ = 10, 30 and 50 g m^−2^, and B) yarns of different composition produced from glycerol plasticized (10% glycerol), deacetylated (40% NaOH) and hybridized (15 gsm chitin‐β‐glucan and 15 gsm NFC) sheets with Γ = 30 g m^−2^.

**Figure 4:**
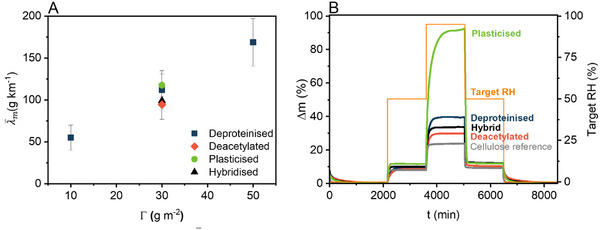
A) Linear density ${\bar{\lambda }}_{m}\;$ of deproteinized chitin‐β‐glucan yarns as a function of grammage Γ; B) water absorption determined by dynamic vapor sorption, mass difference Δ*m* for plasticized, deproteinized, deacetylated chitin‐β‐glucan and NFC‐chitin‐β‐glucan (hybrid) yarns compared to a cellulose paper yarn reference.

Yarns were produced by evenly rolling small strips cut from the produced sheets. Paper yarns produced from chitin‐β‐glucan sheets had cross‐sections ranging from square to oval/circular (**Figure** **3**) and increased in diameter with increasing sheet grammage Γ: 174 ± 8 µm, 217 ± 22 µm, and 446 ± 36 µm for rolled 10, 30 and 50 gsm sheets, respectively. These diameters were considerably larger than those reported for dry‐spun crustacean (shrimp) fibers (13–31 µm)^[^ ^25^ ^]^ but similar to the diameters reported in studies on wet‐spun fungal monofilaments—140–270 µm^[^ ^9b^ ^]^ and 180–200 µm^[^ ^26^ ^]^—and within the same range as cellulose paper yarns (200–1600 µm).^[^ ^27^ ^]^ Glycerol‐plasticized yarns prepared from 30 gsm sheets were slightly larger in diameter (295 ± 15 µm) than untreated deproteinized chitin‐β‐glucan yarns of the same grammage, likely attributable to yarn swelling due to glycerol uptake. Glycerol‐plasticized yarns resembled their deproteinized chitin‐β‐glucan counterparts, exhibiting a fibrous structure. However, yarns prepared from deacetylated sheets exhibited a rougher texture. Separation at the interface between chitin‐β‐glucan and NFC sheets resulted in considerable void content; visible on both the exterior surface and in the cross‐section of the hybrid yarns. Yarn linear density exhibited a strong linear correlation with sheet grammage due to the increased thickness of the used sheet precursor comprising more material per fiber cross‐section area (**Figure** **4**A).

Plasticized chitin‐β‐glucan yarns (2.5 ± 1.3 wt.% glycerol content) absorbed the most water, exhibiting a maximum moisture uptake of 92 wt.% when exposed to 95% RH due to the miscibility of glycerol with water (Figure 4B). Deproteinized chitin‐β‐glucan yarns exhibited a maximum moisture uptake of 40 wt.% at 95% RH. Notably, this moisture content was even higher than that associated with deacetylated fungal yarns (30 wt.%), which was likely attributable to the compositional differences of the two yarns: both have similar chitin content, and although the deacetylated yarns contained more chitosan than the deproteinized yarns, they also contained considerably less β‐glucan. The higher water sorption capacity of the deproteinized chitin‐β‐glucan yarns is possibly caused by the higher content of hydrophilic components, such as a higher amount of β‐glucan.^[^ ^28^ ^]^ Reference commercial cellulose paper yarns exhibited a slightly lower moisture uptake (24 wt.%) than the deproteinized and deacetylated paper yarns. NFC‐chitin‐β‐glucan hybrid paper yarns had a moisture content of 34 wt.% at 95% RH, which was approximately the midpoint between the values associated with their two constituents: neat cellulose and neat chitin‐β‐glucan.

Yarn tenacity increased with sheet grammage for dry yarns produced from deproteinized chitin‐β‐glucan sheets to a maximum of 11.2 cN/tex (134 MPa) at a threshold grammage of 30 g m^−2^, after which no further increase in tenacity was observed (**Table** **2**). This value was ≈50% higher than that associated with the commercial cellulose paper yarn reference, similar to cellulose paper yarn literature values^[^ ^15^, ^27^ ^]^ and values associated with cotton yarns,^[^ ^15^ ^]^ dry‐spun crustacean (shrimp) chitin fibers,^[^ ^25^ ^]^ viscose yarns^[^ ^15^ ^]^ and wet‐spun cellulose fibers^[^ ^25^ ^]^ but lower than those associated with dry‐spun cellulose fibers.^[^ ^25^ ^]^ The initial yarn modulus was independent of yarn linear density. The dry deproteinized chitin‐β‐glucan yarns exhibited an initial modulus of 300–325 cN/tex (3.59 ‐ 3.89 GPa), which remained stable within the error margins across various sheet (yarn precursor) grammages. Deproteinized chitin‐β‐glucan yarns were approximately twice as stiff as the commercial cellulose paper yarn reference, but about half as stiff as dry‐spun crustacean (shrimp) chitin and wet‐spun cellulose fibers and about a quarter as stiff as dry‐spun cellulose fibers.^[^ ^25^ ^]^ ε_*f*_ of dry yarns produced from chitin‐β‐glucan sheets increased with sheet grammage from 3.6% at 10 g m^−2^ to 5.6% at 30 g m^−2^ before plateauing; 50 g m^−2^ exhibited only a marginally higher ε_*f*_ of 6.0%. All chitin‐β‐glucan yarns exhibited lower ε_*f*_ than the commercial cellulose paper yarn reference and viscose yarns^[^ ^15^ ^]^ but were on par with literature values for cellulose paper^[^ ^15^, ^27^ ^]^ and cotton^[^ ^15^ ^]^ yarns and dry‐spun cellulose fibers.^[^ ^25^ ^]^ They also resembled published values for chitinous yarns, such as wet‐spun fungal monofilaments^[^ ^26^ ^]^ and dry‐spun fibers from crustacean (shrimp) chitin.^[^ ^25^ ^]^

**Table 2:** Tenacity *T_s_*, initial modulus *E*, and strain‐to‐failure ε_*f*_ of dry and wet (10, 30, and 50 gsm) deproteinized chitin‐β‐glucan, and 30 gsm glycerol‐plasticized, deacetylated and NFC‐chitin‐β‐glucan hybrid yarns compared to a cellulose paper yarn reference. Literature values are also provided for fungal monofilaments, shrimp chitin fibers, cellulose fibers, cellulose paper yarns, cotton, and viscose.

Yarn	gsm	Dry			Wet		
		*T_s_* [cN/tex]	*E* [cN/tex]	ε_*f*_ [%]	*T_s_* [cN/tex]	*E* [cN/tex]	ε_*f*_ [%]
Cellulose paper yarn (reference)		7.5 ± 0.7	132.6 ± 23.7	11.6 ± 1.4	4.76 ± 0.65	25.06 ± 9.53	22.6 ± 3.0
Deproteinized chitin‐β‐glucan	10	9.4 ± 2.8	326.2 ± 138.8	3.6 ± 1.3	0.89 ± 0.38	0.02 ± 0.004	21.4 ± 3.6
	30	11.2 ± 1.7	312.0 ± 111.2	5.6 ± 1.3	0.51 ± 0.15	0.01 ± 0.003	32.0 ± 5.7
	50	9.3 ± 2.1	301.2 ± 82.3	6.0 ± 1.4	0.72 ± 0.15	0.01 ± 0.01	32.0 ± 4.4
Glycerol‐Plasticized	30	1.8 ± 0.5	58.8 ± 20.6	8.3 ± 2.9	0.63 ± 0.21	0.004 ± 0.004	26.6 ± 6.7
Deacetylated (chitosan)	30	3.0 ± 1.2	222.3 ± 82.8	2.1 ± 0.7	0.65 ± 0.33	0.007 ± 0.002	39.8 ± 7.2
NFC‐chitin‐β‐glucan (hybrid)	30	4.0 ± 0.9	260.2 ± 31.0	2.2 ± 0.9	0.21 ± 0.09	0.001 ± 0.006	19.9 ± 7.1
Reference values from the literature							
Fungal monofilaments (wet spun)^[^ ^26^ ^]^		‐	‐	2.6–7.1	‐	‐	‐
Shrimp chitin fibers (dry spun)^[^ ^25^ ^]^		9–16	600–900	3–8	‐	‐	‐
Cellulose fibers (wet spun)^[^ ^25^ ^]^		≈13	750–850	5‐8	‐	‐	‐
Cellulose fibers (dry spun)^[^ ^25^ ^]^		18–21	1100–1300	7–10	‐	‐	‐
Cellulose paper yarns^[^ ^15^, ^27^ ^]^		5–17	‐	3–5	13–16	‐	6–7
Cotton^[^ ^15^ ^]^		12	‐	5	16	‐	7
Viscose^[^ ^15^ ^]^		14	‐	14	8	‐	14

Glycerol plasticization of 30 g m^−2^ deproteinized chitin‐β‐glucan yarns resulted in a factor 5–6 decrease in initial modulus and tenacity, attributable to disrupted intermolecular bonding between fibrils. Glycerol‐plasticized yarns prepared at this grammage did, however, exhibit much higher ε_*f*_ due to enhanced fiber‐fiber mobility. Glycerol molecules penetrate the material and increase the free volume between fibrils leading to slippage.^[^ ^29^ ^]^ Yarns produced from deacetylated (chitosan) sheets (with reduced glucan content) exhibited a lower initial modulus and a tenacity one‐third of the value associated with deproteinized chitin‐β‐glucan yarns at 30 g m^−2^. These yarns also exhibited half the ε_*f*_ of deproteinized chitin‐β‐glucan yarns of the same grammage. The reduced mechanical properties of yarns produced from deacetylated rather than deproteinized sheets may be attributable to their lower glucan content: the glucan matrix is known to improve fibril‐fibril contact.^[^ ^3a^ ^]^ Higher chitosan content in deacetylated sheets may also disrupt chitin crystalline structures, resulting in a more ductile material.^[^ ^30^ ^]^ Hybrid yarns comprising both deproteinized chitin‐β‐glucan and NFC sheets (15 gsm + 15 gsm) exhibited lower tenacity, initial modulus, and ε_*f*_ than deproteinized chitin‐β‐glucan sheets of the same grammage (30 g m^−2^). Contrary to the hypothesis that NFC sheets would reinforce the yarn structure,^[^ ^31^ ^]^ the mechanical properties of NFC sheets fell short of expectations since consolidation and activation steps would be required to achieve the expected mechanical properties. However, consolidated NFC nanopapers were difficult to process into yarns and provided low inter‐sheet adhesion during rolling. Poor interfacing and delamination phenomena also prevented successful load transfer between the sheets in the hybrid yarns.

All wet yarns produced from deproteinized chitin‐β‐glucan sheets had an initial modulus <0.02 cN/tex. They also exhibited tenacities <1 cN/tex, which was one‐fifth of the value associated with the commercial cellulose paper yarn reference and much lower than the tenacity of wet cotton and viscose yarns.^[^ ^15^ ^]^ These poor properties are attributable to water molecules absorbed within the yarn, which disrupt hydrogen bonding between fibrils. Wet yarns did, however, exhibit ≈5 times higher ε_*f*_ than their dry equivalents. These values were equivalent to or greater than those associated with the commercial cellulose paper yarn reference and literature values for wet cellulose paper yarns, cotton and viscose yarns.^[^ ^15^ ^]^ It is, however, worth noting that fair comparison of these yarns is complicated by differences in their construction, such as a number of twists per unit length, linear density and spinning method, in addition to differences in the properties of the fibers from which they are produced, e.g., fiber material, length and linear density.

Glycerol‐plasticized yarns exhibited the highest wet stiffness at a similar tenacity to deproteinized chitin‐β‐glucan yarns but a lower ε_*f*_. Glycerol promotes higher dimensional stability in yarns by hydrogen bonding formation with chitin to form networks.^[^ ^32^ ^]^ Wet yarns produced from deacetylated sheets were also stiffer than those produced from deproteinized chitin‐β‐glucan sheets of the same grammage (30 g m^−2^). These higher mechanical properties are attributable to the lower glucan content of deacetylated yarns, and hence lower water absorption (30 wt.% compared to 40 wt.%). Wet hybrid chitin‐β‐glucan/NFC yarns exhibited a similar behavior: increased initial modulus compared to wet deproteinized chitin‐β‐glucan yarns due to lower water absorption (34 wt.% compared to 40 wt.%).

## 3 Conclusion

We present a sustainable method for manufacturing chitinous fungal yarns based on the ancient Japanese art of Shifu. Deproteinized chitin‐β‐glucan yarns are stiffer and stronger than commercial cellulose paper yarns but have lower strain to failure. Tensile strength increased with grammage, peaking at a threshold grammage of 30 gsm. To state the obvious: the properties of yarns correlate with the properties of the sheets from which they are made, i.e., to make better yarns, use better sheets. Various chemical modifications also facilitate tuning of yarn mechanical properties: plasticizing chitin‐β‐glucan yarns with glycerol resulted in increased strain to failure at the cost of stiffness and strength due to improved fibril slippage. Contrary to expectations, hybridizing chitin‐β‐glucan sheets with NFC sheets during yarn rolling resulted in a reduction in yarn stiffness, strength, and strain to failure due to the high porosity of the unconsolidated NFC sheets and poor inter‐sheet interfacing in the yarns.

Fungal chitin‐β‐glucan yarns, exhibiting mechanical properties similar to chitin and commercial cellulose paper counterparts, offer a biobased alternative to synthetic yarns. The Shifu technique and paper yarn spinning methods represent a remarkable way to convert hugely popular but very application‐limited mats of fungal biomass into much more versatile yarns that can be spun for a broad range of applications. This could allow textile fungal biorefineries to become a resource‐efficient and scalable route for upcycling of agricultural waste products into spun and woven textiles and help them achieve widespread adoption.

## 4 Experimental Section

### Materials

*Agaricus bisporus* (white button) mushrooms were purchased from a local convenience store (origin: B. Fungi Kft., Ocsa, Hungary). NaOH (≥97.0%) and glycerol 1.26 were purchased from Wilhelm Neuber's Enkel Dr Brunner & Kolb GmbH. Nanocellulose was produced from a 3 wt.% beech wood pulp suspension in water, ground for 10 cycles using a disc mill (Granomat JP 150). Commercial paper yarn was purchased as a reference from PaperPhine (Vienna, Austria). Sugar recovery standards (SRS) for carbohydrate analysis were prepared from L‐(+)‐arabinose (Calbiochem), D‐(+)‐galactose (Merck), D‐(+)‐glucosamine hydrochloride (Sigma‐Aldrich), D‐(+)‐glucose (BDH Prolabo), D‐(+)‐mannose (Merck), L‐(+)‐rhamnose (BDH Prolabo) and D‐(+)‐xylose (Merck). All chemicals were used as received. Deionised water was used for all experiments.

### Extraction of Polymer Complexes From the Mushrooms (Deproteinization) and Deacetylation

Deproteinization was adapted from the process established by Nawawi et al.^[^ ^21b^ ^]^ *A. bisporus* fruiting bodies were washed twice in water (1 kg L^−1^) and blended in batches for 3 min per batch (Tristar, BL‐4473 VitaPower 2000). The resulting slurry was then heated to 85 °C for 30 min, cooled to 25 °C and filtered over a linen cloth. The supernatant was removed, and the precipitate suspended in a 1 m NaOH solution for 3 h at 65 °C. The suspension was then cooled to 25 °C and neutralized (pH 7) by repeated washing with water over a linen cloth, yielding deproteinized chitin‐β‐glucan polymer extract.

Deacetylation of deproteinized chitosan‐β‐glucan polymer complex was inspired by the literature producing chitosan from marine chitin sources.^[^ ^33^ ^]^ Partial deacetylation was achieved in 12 m (40% w/v) aqueous NaOH at 100 °C for 1 h. The suspension was then neutralized (pH 7) by repeated washing with water over a linen cloth.

### Analysis of the Molecular Structure of the Polymer Complexes

Solid state nuclear magnetic resonance spectroscopy (ssNMR) was conducted on a Bruker AVANCE NEO 500 MHz wide‐bore system using a 2.5 mm magic angle spinning probe. The resonance frequency for ^13^C NMR was 125.78 MHz and the MAS rotor spinning was set to 14 kHz. Cross‐polarization was achieved by a ramped contact pulse with a contact time of 1 ms. During acquisition, ^1^H was high power decoupled using SPINAL with 64 phase permutations. The chemical shifts for ^13^C are reported in ppm and are referenced externally to adamantane by setting the low field signal to 38.48 ppm.

C, H, N, S, and O elemental analysis was completed for triplicate 2 mg samples using a EuroVector EA 3000 CHNS−O elemental analyzer and a TraceDec conductivity detector. Carbohydrate analysis was conducted by high‐performance anion exchange chromatography (HPAEC). A 300 mg freeze‐dried sample was mixed with 3 mL of 72% sulfuric acid at 30 °C for 60 min. The acid was then diluted with water to a 4% concentration, and the mixture was placed in an autoclave at 121 °C for 60 min. HPAEC was performed on the previously diluted acid hydrolase using a Dionex ICS3000 chromatograph equipped with a CarboPac PA20 column. Sugar recovery standards were prepared and pretreated under identical hydrolysis conditions prior to HPAEC analysis to analyze their recovery throughout the procedure.

The fraction of acetylated monosaccharide units (*X_acetyl_*) was calculated as the average value of CH_3_ (22.5 ppm) and C═O (173.5 ppm) ssNMR peak integrals (expressed as a percentage). The degree of acetylation (*DA*) was calculated based on *X_acetyl_* and the glucosamine content (relative to the total sugar content) as measured using HPAEC (Equation 1). Chitin (Equation 2) and chitosan (Equation 3) content were calculated using the *DA* and glucosamine content. Glucan content was equivalent to glucose content (relative to the total sugar content) as measured using HPAEC.

(1) $$DA\;\left(\%\right)=\frac{X_{acetyl}}{\left[glucosamine\right]}$$

(2) $$Chitin\;content=DA*\left[glucosamine\right]$$

(3) $$Chitosan\;content=\left(100-DA\right)*\left[glucosamine\right]$$

FTIR was performed using a Tensor II FTIR spectrometer (Bruker). IR spectra were obtained for the range 500–4000 cm^−1^ with a resolution of 4 cm^−1^. Spectra were recorded using the integrated OPUS 7.5 software.

All wide angle X‐ray diffraction reflection data were recorded on a PANalytical Empyrean Powder Diffractometer in theta/theta mode on a reflection‐transmission spinner in reflection mode using a Bragg‐Brentano mirror. The anode material was Cu, and the measurement temperature was 22 °C. All measurements are continuous scans in the range of 3–65° in 2Θ, with a step size of 0.029° and a count time per step of 2021 s. All transmission data were measured on a Xenocs Nano‐inXider. The low‐angle range was from 0.005–5.2° (q‐range from 0.00037 to 0.37 Å^−1^) and the wide‐angle range was from 4.11–62° in 2Θ. The counting time was 30 times 600 s for both wide and low angles simultaneously.

### Preparation of Sheets from Polymer Complexes

Pre‐determined quantities: 0.11, 0.33, and 0.55 g dry mass of deproteinized and deacetylated chitin−β‐glucan polymer extracts were suspended in 300 mL of water and mechanically blended for 3 min (Tristar, BL‐4473 VitaPower 2000) to produce 10, 30 and 50 gsm sheets, respectively. We determined the dry mass content of the fungal pulp in suspension by weighing pre and postdrying and used a defined mass to prepare each sheet, resulting in grammages that match our target grammage within acceptable experimental error (±10% of intended grammage). The suspensions were then vacuum filtered (VWR 413, qualitative filter paper, particle retention 5–13 µm), the filter paper peeled off and the wet filter cakes cold pressed for 5 min between blotting papers to remove any excess moisture. We did not observe any visible loss of material during the filtration step. Fungal pulp is brown in color and thus would be visible when passing through the filter paper. The sandwich of wet filter cake and blotting papers was pressed between two metal plates under a 5 kg‐force with blotting papers exchanged twice during this process. The extracts were then dried at 65 °C for 8 h between baking paper‐lined metal plates under a 5 kg‐force. NFC sheets (30 gsm) were produced in the same way except that they were not (hot or cold) pressed but simply sandwiched between two metal plates to prevent wrinkling during drying. This drying method was adopted because once pressed, the NFC sheets did not adhere to the chitin‐β‐glucan sheets, making hybridization impossible.

### Physical and Mechanical Analysis of the Sheets

Sheets were cut into dumbbell‐shaped specimens (shape according to type 1BB, BS EN ISO 527‐2, 2012) using a Zwick ZCP 020 manual cutting press. Specimens had a parallel width of 5 mm, a parallel length of 58 mm and an overall length of 75 mm. The thickness and mass of each specimen were determined using an (Anyi Measuring) digital outside micrometer and an analytical microbalance (OHAUS Explorer). The envelope density ρ_*e*_ (associated with the entire volume of the material including both solid material and internal voids and pores) of the sheets was calculated as the ratio of the mass *m* over the surface area *A* of the specimen, and thickness *t* (Equation 4).

(4) $$\rho_{e}=\frac{m}{A\;\cdot \;t}$$

Tensile tests were performed using a dual‐column universal testing system (model 5969 Instron) equipped with a 1 kN load cell and a noncontact video extensometer (Gig ProE iMETRIUM). Specimens were fixed between metal clamps and blotting papers used to avoid perforation. Testing velocity was 1 mm min^−1^ with a gauge length set to 25 mm. Elastic moduli *E* were determined in the linear elastic region as a secant between stress values separated by 0.2% strain. The sheet tensile strength σ was calculated using the maximum load over the specimen cross‐sectional area.

Sheet skeletal density ρ_*s*_ (associated with the solid material volume of the material, only excluding internal voids or pores) was analyzed using a helium gas displacement pycnometer (Micromeritics AccuPyc II 1340) with a 1 cm^3^ chamber for ten replicates. The porosity Φ was then calculated from the measured skeletal and envelope densities (Equation 5).

(5) $$\Phi =1-\frac{\rho_{e}}{\rho_{s}}$$

### Preparation and Plasticization of Paper Yarns

The wet filter cake was cut into strips with a width of 2 mm using a scalpel blade and then rolled gently by hand, moving the fingers from the center to the extremities of the yarn

Dry deproteinized chitin‐β‐glucan yarns were soaked for 24 h in 10% glycerol to plasticize them. The yarns were hung under the tension delivered by a 20 g weight until completely dry.

Hybrid yarns were prepared by 1) producing both a 15‐gsm chitin‐β‐glucan sheet and a 15‐gsm NFC sheet, 2) cutting the wet filter cakes into strips, 3) lying an NFC strip on top of a chitin‐β‐glucan strip and finally 4), rolling the two into a yarn while still wet. The resulting hybrid yarns were dried for 5 min while under tension delivered by a 20 g weight.

### Morphological Analysis of Paper Yarns

Imaging of the paper yarns was performed using scanning electron microscopy (NeoScope JCM‐7000, Jeol, Japan). An accelerating voltage of 30 kV was used. All samples were coated with gold prior to imaging (Fine coater JFC‐1200, Jeol, Japan).

### Physical and Mechanical Analysis of the Paper Yarns

Moisture sorption behavior was investigated using dynamic vapor sorption (DVS Intrinsic, Surface Measurement Systems, London, UK). Samples were exposed to 0%, 50% and 95% RH, for 12 h periods. All measurements were run at 25 °C and the change in mass Δ*m* resulting from moisture sorption was measured as a function of time. The glycerol content was determined using the DVS Intrinsic (Surface Measurement Systems, London, UK). Plasticized yarns (30 g m^−2^) with lengths between 9.4 cm and 9.7 cm were dried at 0 RH% for 27 h to determine the remaining water content within the yarn. The difference between linear density between dried plasticized and unplasticized yarns was attributed to the glycerol content. Samples were measured in triplicate.

The linear density ${\bar{\lambda }}_{m}$ was calculated based on the mass *m* (Satorius Microbalance) and the yarn length *l*, measured using a caliper (Equation 6):

(6) $${\bar{\lambda }}_{m}=\frac{m}{l}$$

Tensile tests were performed on dry paper yarns using a dual‐column universal testing system (model 5969 Instron) equipped with a 1 kN load cell and the actual strain measured using a noncontact video extensometer (Gig ProE iMETRIUM). Specimens were fixed between paper mounting tabs with a length of 40 mm and a width of 30 mm using epoxy glue. Testing velocity was 0.5 mm min^−1^ with a gauge length set to 10 mm.

Tensile tests were performed on wet paper yarns (soaked in distilled water for 5 min prior to testing) using a single‐fiber tensile testing machine (FAVIMAT+, Textechno, Germany) with a 200 cN loadcell. Testing velocity was 1 mm min^−1^ (to prevent drying during testing) with gauge length set to 10 mm.

Tenacity *T_s_* (cN/tex) was calculated based on the force at break and the linear density. The initial elastic modulus *E* was analysed in the linear elastic region as a secant between stress values separated by at least 0.2% strain.

## Conflict of Interest

The authors declare no conflict of interest.

## Supporting information



Supporting Information

## Data Availability

The data that support the findings of this study are openly available in zenodo.org at https://10.5281/zenodo.16316012, reference number 16316012.
