# Supplementary material for: Shifu‐Inspired Fungal Paper Yarns
Source: Adv Sci (Weinh). 2025 Aug 4;12(40):e11975. doi: 10.1002/advs.202511975 (PMC12561255; doi:10.1002/advs.202511975)
Supplement: Supplementary file 1 — Supporting Information [file ADVS-12-e11975-s001.pdf]

Electronic supplementary material for:

Shifu-inspired fungal paper yarns

Anne Zhao[a], Mitchell P. Jones[a], Kathrin Weiland[a,b,c]* & Alexander Bismarck[a,c,d]*

[a] Institute of Material Chemistry and Research, Polymer and Composite Engineering (PaCE) Group, Faculty of Chemistry, University of Vienna, Währinger Straße 42, 1090, Vienna, Austria
[b] Shaping Matter Lab, Faculty of Aerospace Engineering, Delft University of Technology, 2629 HS Delft, Netherlands.
[c] Department of Chemical Engineering and Ecotoxicology, University of Applied Sciences Technikum Wien, Vienna, Austria
[d] Division of Materials Science, Department of Engineering Sciences and Mathematics, Luleå University of Technology, SE-97187 Luleå, Sweden
* Corresponding author. *Email address*: kathrin.weiland@technikum-wien.at (K. Weiland), alexander.bismarck@univie.ac.at (A. Bismarck).

List of Figures

Figure S1. SEM images of the surface of deprotonised chitin-glucan sheets (30 g/m2) showing collapsed fungal cell walls (left), which act as a carrier for nanofibrils (middle) and cross-section (right).

Figure S2. ATR-FTIR spectra of deproteinised (blue), deacetylated (red) and plasticised (green) yarns at a) 3356 cm^{-1}, bands associated with O-H b) 1350 to 1750 cm^{-1}, bands associated with –C=O and N–H and c) 1026 to 1150 cm^{-1}, bands associated with C-OH contained in structural biopolymers.

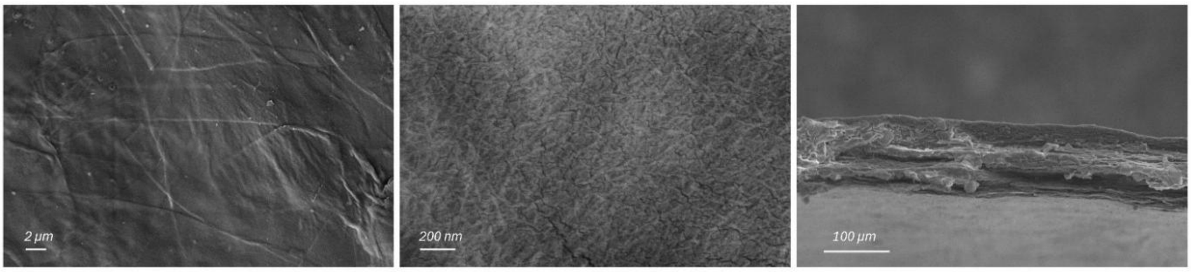

Figure S1. SEM images of the surface of deprotonised chitin-glucan sheets (30 g/m^2) showing collapsed fungal cell walls (left), which act as a carrier for nanofibrils (middle) and cross-section (right).

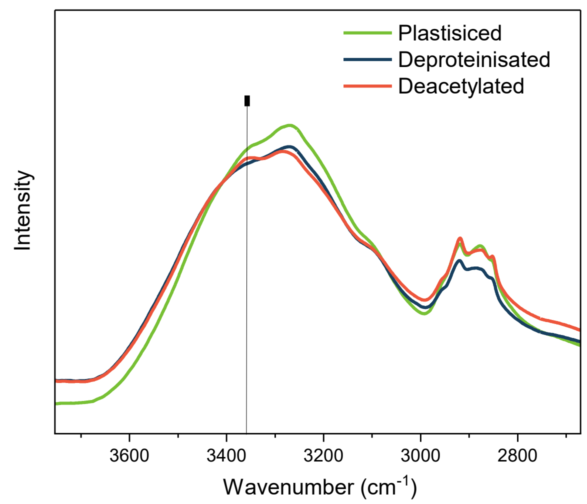

(a)

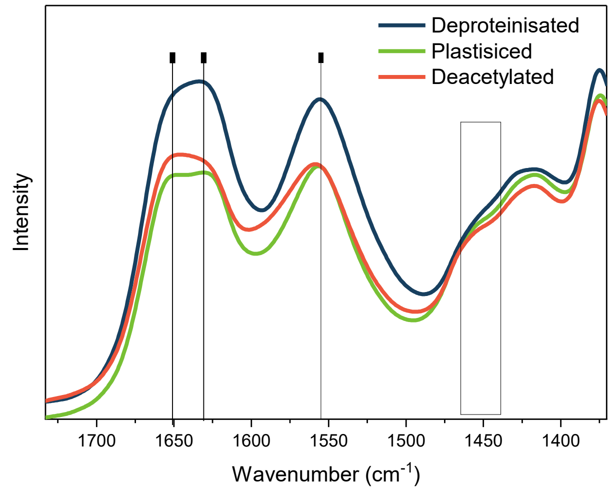

(b)

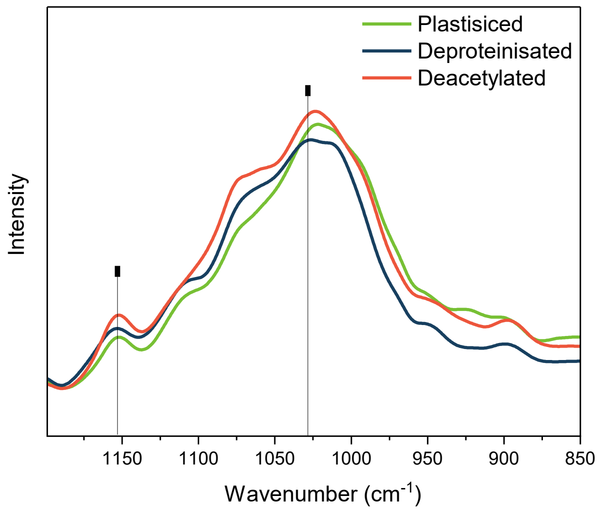

(c)

Figure S2. ATR-FTIR spectra of deproteinised (blue), deacetylated (red) and plasticised (green) yarns at a) 3356 cm^{-1}, bands associated with O-H b) 1350 to 1750 cm^{-1}, bands associated with –C=O and N–H and c) 1026 to 1150 cm^{-1}, bands associated with C-OH contained in structural biopolymers.
